# Effects of Microplastics on Selected Earthworm Species

**DOI:** 10.3390/toxics13030201

**Published:** 2025-03-11

**Authors:** Marek Klimasz, Anna Grobelak

**Affiliations:** Faculty of Infrastructure and Environment, Czestochowa University of Technology, 42-201 Czestochowa, Poland; anna.grobelak@pcz.pl

**Keywords:** microplastic, high-density polyethylene, adaptive response, earthworms

## Abstract

Microplastics currently pose a serious threat to aquatic and terrestrial ecosystems. The high mobility of particles and their diversity in size, material and shape lets them spread widely. Further complicating matters is the ever-expanding plastics industry and modifications to its manufacturing processes. To date, many cases of negative, often toxic effects of microplastics on various species such as fish, birds and mammals have been documented. The methodology for measuring and determining the effects of microplastics on soil organisms is still an area of little understanding and certainly requires further study. In our conducted experiment, we reported the effects of selected microplastics in soil (polyethylene, polyethylene terephthalate, polystyrene, polyamide and a mixture of these plastics) at concentrations of 0.1% *w*/*v* and 1% *w*/*v* at two time intervals, one and three months, on five different earthworm species, identifying the species-related microplastic response. This study investigated the effects of different microplastics on biological parameters such as survival and respiration and biochemical parameters such as effects on glutathione s-transferase (GST), a marker of detoxification and adaptive response in earthworm species *Eisenia andrei*, *Eisenia fetida*, *Lumbricus terrestris*, *Apporectoda caliginosa* and *Dendrobena veneta.* The choices of species and the types of microplastic selected are intended to map the occurrence of microplastic contamination in the soil and determine the adaptation of earthworms to changing environmental conditions, considering their ecological significance and functional diversity in soil ecosystems.

## 1. Introduction

Earthworms are a widespread group of animals inhabiting almost all continents, including the Holoarctic area extending through Europe, the northern part of Africa, North America and most of Asia; species adapted to prolonged frosts can also be found in areas of present-day Siberia [1]. Because of their widespread environmental adaptation, some of them are referred to as cosmopolitan, that is, commonly found in large areas spanning different countries and often continents [2]. Their natural habitat is nutrient-rich and organic matter-rich soils with a pH in the range of 5–8, but species are known to prefer forested areas with much lower pH values, as well as sandy and calcareous areas with values of pH 8. Soil is the natural habitat and breeding ground for the vast majority of earthworm species, and their presence is extremely important from a soil ecosystem perspective [3]. Their species diversity and preferred foods make it possible for us to classify them into many categories of ecosystem usefulness such as bioindication, composting or the assessment of particular soil contaminants [4]. Under favorable conditions, their reproduction rate is high, and factors categorized as growth inhibitors include heavy metals, pesticides and prolonged drought. The presence of earthworms in the soil, the tunnels they dig and the mucus they secrete affect the amount of oxygen in the soil, the soil structure and the chemical composition of soil aggregates [5]. In addition, earthworms’ activities lead to changes in the microbial environment, which are associated with an increase in the metabolic productivity of the soil. It is also necessary to mention the function associated with the decomposition of organic matter, both directly (use as food) and indirectly (use by soil microorganisms of compounds excreted by earthworms in the form of feces or mucus). This phenomenon is commonly used in vermicomposting, a process of valorizing organic matter, the product of which is humus that is valuable to plants [6].

Plastics, most commonly polymers, are widely used in everyday life due to their strength, ease of molding, resistance to physical factors and low production costs [7,8]. Globally, the amount of microplastics (MPs) in soils is estimated to be between 1.5 and 6.6 million tonnes, with the heaviest contamination reported in China, where approximately 660 kilotonnes were released into the soil [9]. The nomenclature of microplastics is still a debatable topic due to the methods of sampling and different measuring apparatus [10]. There is a pervasive concern that MPs originating from bigger items or can be produced as primary MPs and end up in ecosystems, affecting biota and their functioning and also posing a risk to humans. Plastic production has been increasing exponentially since the 1950s, with the expected annual production rate estimated at 1100 tonnes by 2050 [11].

Research on microplastics (MPs) has mainly focused on aquatic ecosystems, while studies on their impacts on terrestrial environments remain relatively scarce. Recent reviews, such as that by Kallenbach et al. [12], emphasize that although MPs are well documented in marine and freshwater systems, their fate, transport and impact on soils and terrestrial organisms are still under-researched. Similarly, the recent review by [9] emphasizes the critical knowledge gaps in understanding MPs’ impact on soil ecosystems, particularly their effects on microbial communities, nutrient cycling and soil structure. The review by Kallenbach et al. [12] emphasizes that MPs in terrestrial systems may pose unique risks due to their interactions with soil biota, aggregation in the soil matrix and potential for long-term accumulation.

Microplastics (MPs) can affect various aspects of terrestrial ecosystems, altering soil properties, affecting microbiome composition and influencing the motility, growth rate and reproductive capacity of soil animals. The impact of MPs on plants depends on their size, shape and polymer type, as well as on exposure time and concentration, and they can both impair growth and modify soil properties, which indirectly affect MPs’ toxicity. In the case of soil organisms such as earthworms, MPs are transported and incorporated in the soil through processes such as bioturbation, which can increase their toxicity. In particular, nanoplastics (NPs) exhibit higher levels of toxicity than MPs, and NP concentrations above 1 g/kg can lead to growth inhibition and reduced survival in earthworms [9]. The plastics produced so far undergo physical and chemical fragmentation processes, during which mechanical friction, UV radiation or water can affect the release of microparticles, the concentration of which increases every year [13]. Estimating the actual concentration of these particles in soil is difficult for several reasons: the geological variation in the soil (pH, density, amount of water, etc.); the accuracy of the apparatus, which is not always capable of detecting all plastic particles; and the reproducibility of the samples. Studies conducted in many parts of the world describe the concentration of microplastics in relation to dry matter at the level of 0.001 to 0.025 mg/kg. The results of studies conducted in East Asian countries indicate that highly urbanized cities, often without adequate sewage infrastructure, are characterized by the presence of microplastics in the soil at levels of 0.25–0.55 mg/kg which seems to be an extreme result [14]. It should be noted that plastics are classified as micropollutants, that is, substances and compounds in trace amounts that are measurable and have a negative impact on the ecosystem.

Studies have shown that microplastics can negatively affect organisms at both the individual and population levels. Their presence in the gastrointestinal tract can cause physical damage to soft tissues and lead to allergic reactions or poisoning by heterogeneous toxic compounds [15]. In addition, microplastic contents in the intestines and stomach can cause digestion disruption or a sense of false satiety and reduce the amount of substances absorbed by the body [16,17]. Consequently, this translates to slower growth of the body [18].

The purpose of this study was to determine the effects of different microplastics at two different concentrations on selected earthworm species. The study was designed based on biological assessments of natural immune responses and morphological changes due to contamination contained in the soil. For this purpose, the species used were *Eisenia fetida*, *Eisenia andrei*, *Lumbricus terrestris*, *Dendrobena veneta* and *Apporectoda caliginosa.* These species represent different ecological groups: epigeic (*E. andrei*, *E. fetida*), anecic (*L. terrestris*) and endogeic (*A. caliginosa*, *D. veneta*), each contributing to organic matter decomposition, soil aeration and structure formation. Their presence influences nutrient cycling, microbial activity and soil stability, making them essential indicators of soil health. Including these species allows for a comprehensive assessment of microplastic impacts across various soil ecological niches. The results show the effects of microplastics on individual survival, mass correlation over time and effects on oxidative stress and respiration.

## 2. Materials and Methods

### 2.1. MP Preparation for Experimentation

This study used microplastics with different chemical structures and fraction sizes for the maximum reproduction of natural urban environmental pollution: fluorescent green polyethylene (PE) microspheres at sizes of 1–5 µm, 10–20 µm, 32–38 µm, 38–45 µm and 53–63 µm; purple polyethylene microspheres at 1.00 g/cm^3^ and sizes of 38–45 µm and 75–90 µm; polyethylene terephthalate (PET) in the form of irregular granules measuring 10–100 µm in size; polyamide (PA) in the form of fibers 10–2000 µm in length; and polystyrene (PS) in the form of irregular film shreds 20–100 µm in size (Cospheric, PO Box 636. Somis, CA 93066, USA). Considering the current levels of pollution and the continuous yearly increase in microplastic concentrations in the soil, it was decided to use two concentrations at a decimal interval, i.e., 0.1% *w*/*v* and 1% *w*/*v*. A weight of 30 g of microplastic was used for 3 L of soil at a 1% (*w*/*v*) concentration and 3 g of microplastic was used for 3 L of soil mixture at a 0.1% (*w*/*v*) concentration, with a soil density of 1.3 kg/L. The 1% *w*/*v* value is intended to show both the impact of extreme contamination on organisms and the possible prevention of these effects, as well as the possibility of detecting microplastics through the calibration of the measurement apparatus.

### 2.2. Experiment Design and Settings

Despite obvious similarities related to the appearance of earthworms, differences within species are significant and include individual size, body pigmentation, preferred environment, foraging mode and sensitivity to individual stresses. Thus, for this experiment we used five earthworm species: *Eisenia andrei*, *Eisenia fetida*, *Lumbricus terrestris*, *Apporectoda caliginosa* and *Dendrobena veneta.*

For biochemical tests, natural agricultural soil was used, mixed with horse manure at a ratio of 1/5 and with microplastics by dry matter conversion. The earthworms used in the laboratory test were self-cultured from a strictly controlled culture (temperature 20 ± 2 °C; photoperiod 16 h L:8 h D) and had similar weights of 0.3 g (±0.02) when placed in the experimental containers. Containers of 3 L contained 10 earthworms or, in the case of *L. terrestris*, 5 per container. For each species, two experimental and one control containers were prepared. After a period of 1 month, the earthworms were taken from the soil, washed thoroughly with deionized water and transferred to sterile agar medium to remove the gut contents. For this experiment, 5 earthworms from each sample were subjected to deep-freezing temperature shock at −80 °C, and then the tissue fragments were homogenized. The remainder were used to map microplastic particles in the tissues. Measurement results for each sample were averaged.

### 2.3. Biotic Response of Earthworms to MPs 

Glutathione s-transferase (GST) is included in an extensive group of proteins commonly found in both animals and plants, prominent in processes related to stress tolerance responses induced by biotic and abiotic factors such as, in the case of animals, lack of food, temperature or the presence of toxins. They are located in both the intercellular space and the cytosol and are integral to the assessment of environmental stress. The main function of s-transferase is the coupling (catalytic) of electrophilic synthetic compounds or those of natural origin to glutathione, and the course of the reaction can be illustrated by the following equation:

GSH + RX → GSR + HX

Many classes of transferases have been described taking into account specific amino acid similarities, as well as the position and number of introns. These classes include Phi, Zeta, Omega, Tau and Lambda, but the literature reports that more can be added.

The method relies on the coupling reaction of reduced glutathione (GSH) with 1-chloro-2,4-dinitrobenzene (CDNB) catalyzed by glutathione S-transferase contained in animal cells. The activity of the enzyme is expressed by measuring the concentration of the resulting complex, i.e., 2,4-dinitrophenyl-S-glutathione. Among the markers of oxidative stress, we additionally distinguish catalase (CAT) and lipid peroxidation determined by quantification via the thiobarbituric acid reaction (TBARS). This methodology has been described in detail by Habig [19] and Aebi [20]. The determination of the change in protein concentrations as a consequence of oxidative stress was further described by [21].

Individuals in the control and test groups, after being separated from the soil, were washed thoroughly with distilled water and placed on sterile agar medium to expel the intestinal contents. Ten specimens of similar weights were placed in each chamber for a period of 24 h at a CO_2_ zero starting point. The study used an Echo respirometer with a 12-channel chamber for 2.8 L cylindrical solid fraction tanks and the following parameters: O_2_ sensor range: 0–25%, accuracy: 0.2%; CO_2_ sensor range: 0–2000 ppm, accuracy: 0.2% (sensors combined). An air pump was used, and controlled measurements were taken from the sealing of the circulation for the value of 10 individuals of species in a 24 h period of bulk measurement and flow measurement. Indicator CO_2_ concentration was determined, and data were compiled in Excel after being synchronized with measuring chambers. Analysis was performed for a mixed microplastic fraction. The respirometric tests were prepared under standard methods: ISO 16072:2002—Soil quality—Laboratory methods for determination of microbial soil respiration and ISO 17155:2012—Soil quality—Determination of abundance and activity of soil microflora using respiration curves.

This study compared the effectiveness of bacterial flora growth on selected media for each type of microplastic and selected earthworm species. LB agar for total bacteria number estimation (Luria broth; ISO 4833-1:2013) and selective MacConkey medium for differentiating lac+ (purple) *Escherichia coli* and *Klebsiella* from lac− (white) *Salmonella* and *Shigella* were used (9 ISO 21528-2:2017). The bacterial flora was collected from the cleaned fresh intestine of the test species and incubated at 37° for 48 h using the spreading plate method.

### 2.4. Data Analysis

One-way analysis of variance (ANOVA) was performed, followed by Tukey’s test as a post hoc analysis. The asterisks in the figures indicate groups for which a statistically significant difference in GST activity was found compared to other groups. The differences may concern both the effect of different types of microplastics (PE, PET, PS, PA, Blend) and the effect of concentration (0.1% vs. 1%).

## 3. Results

### 3.1. Effect of Microplastics on Glutathione S-Transferase

**Figure 1:**
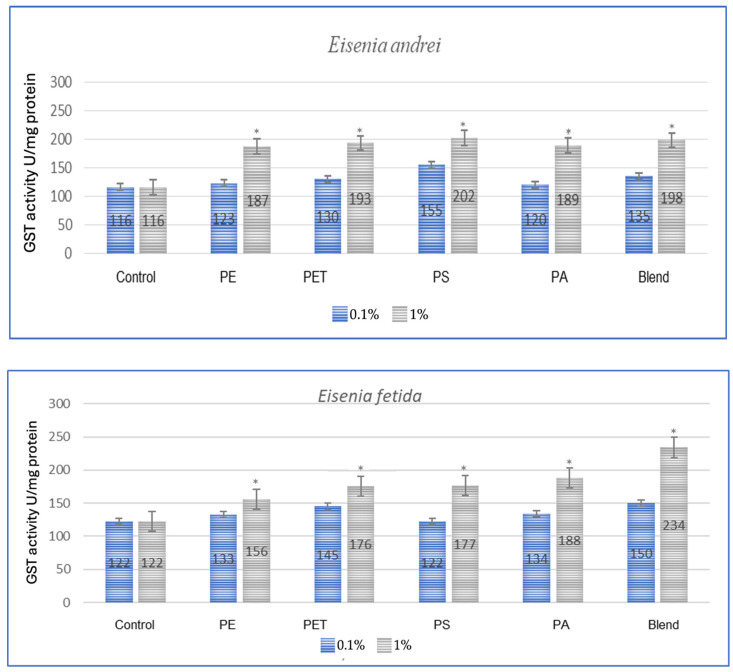

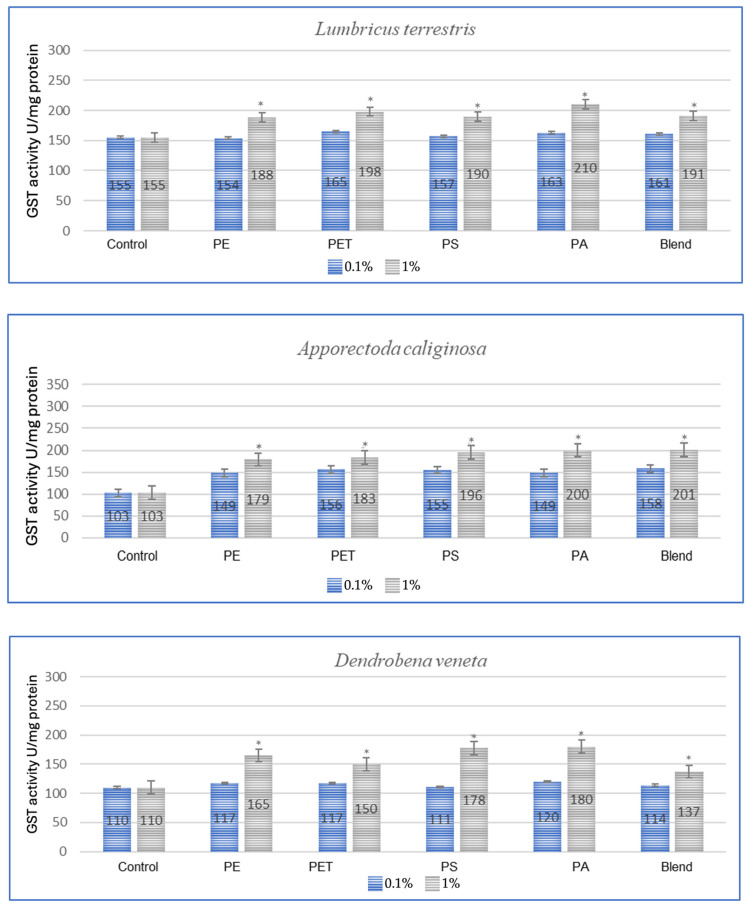
Effect of microplastics on GST activity after 1 month of exposure in tested earthworms; statistically significant differences marked with “*”.

**Figure 2:**
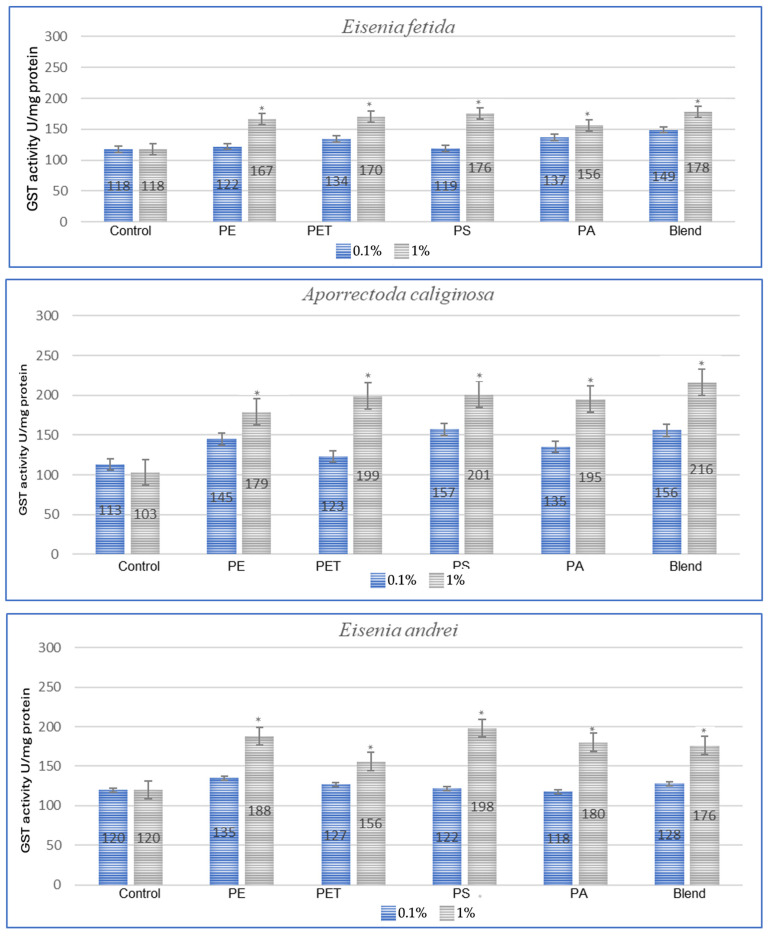

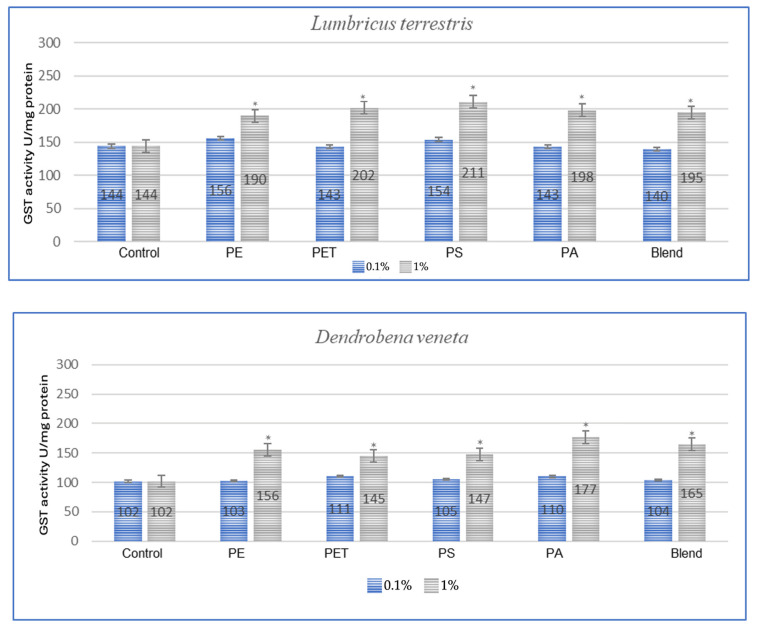
Effects of microplastics on GST activity after 3 months of exposure in tested earthworms; statistically significant differences marked with “*”.

The effect of microplastics on adaptive biomarkers, as indicated by the increase in glutathione S-transferase (GST) concentration, was evident in all studied species. Both after a one-month period, as shown in Figure 1, and after three months, as observed in the same individuals in Figure 2, the changes were evident.

All tested earthworm species showed increased GST activity after exposure to microplastics, which confirms the oxidative stress caused by them (Figure 1). The applied MP blend induced the strongest effect on *E. fetida* and a slightly weaker effect on other species, which suggests differences in detoxification. PA (polyamide) and PS (polystyrene) were the most toxic, causing a significant increase in GST activity in *E. andrei*, *A. caliginosa*, *L. terrestris* and *D. veneta*. In the conducted test, the species *E. fetida* and *L. terrestris* showed the greatest susceptibility to oxidative stress, which means that they may be better bioindicators of MP contamination. Additionally, the species *D. veneta* showed the smallest change in GST after exposure to the MP blend, which may suggest a different detoxification strategy or lower MP absorption.

In the studies conducted after 3 months of exposure (Figure 2), compared to 1 month for *E. fetida*, the strongest effect is maintained for PET and PS, which suggests that long-term exposure to these polymers causes persistent oxidative stress. For *E. andrei*, compared to the results at 1 month, PS and PA groups show even greater GST activity, which suggests the accumulation of the toxic effects of these polymers over a longer period. For the species *A. caliginosa*, compared to 1 month, most polymers show even stronger effects, especially the blend of MPs and polyamide, which suggests the accumulation of toxic effects. In the studies on *L. terrestris*, it was shown that compared to the results after 1 month, most GST values increased, which suggests a gradual accumulation of toxicity and stress. For *D. veneta*, compared to 1 month, the effect of microplastics on GST in this species is more pronounced, especially for PA and Blend, which indicates a gradual increase in stress.

### 3.2. Effect of Microplastics on Respiration

This is one of the first documented studies on the effects of microplastics of different fractions on the respiration of earthworms exposed to microplastics. Previously described studies included the effects of heavy metals or other types of pollutants, and respiration results were reported in μm/L O_2_/g^−1^/h^−1^. Gas exchange in earthworms occurs through their richly vascularized body shells, and diffusion involves the intraepithelial capillary network. Celomatic fluid and mucus further facilitate respiration and the intracellular distribution of oxygen, which, after entering the blood, is distributed throughout the body. Another adaptation to soil life is dissolved hemoglobin in the blood, allowing these animals to survive periodic oxygen shortages. Considering the distribution of microplastics and their ability to hook onto the pores of the body, a question was raised regarding the effect of microplastics on the respiration of individuals.

**Figure 3:**
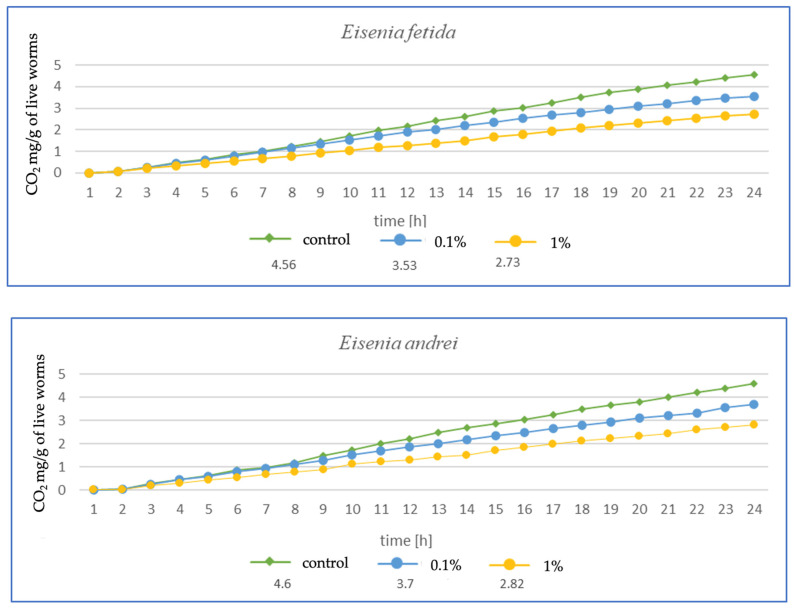

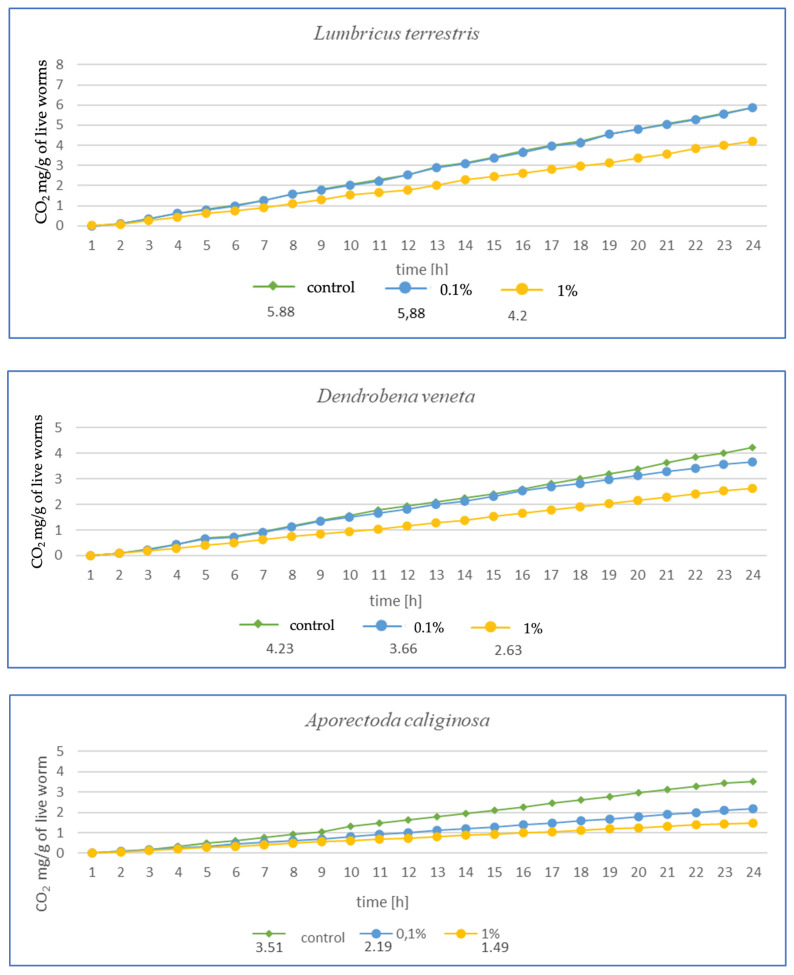
Effects of microplastics on respiration after 1 month of exposure in tested earthworms; mg CO_2_ emitted per g of live worms and per hour.

The higher the MP concentration in *E. fetida*, the greater the reduction in CO_2_ mg emitted per g of live worms, suggesting a decrease in metabolic activity (Figure 3). Additionally, the respiration rate was significantly lower in the 1% MPs group, which may indicate a negative impact of microplastics on the metabolic capacity and health of earthworms. In *E. andrei*, a trend similar to that in *E. fetida* was observed, although the decrease in respiration was somewhat less dramatic, and lower CO_2_ emissions at higher MP concentrations suggested a reduction in metabolic activity. In tests for *L. terrestris*, no effect of MPs on respiration was noted at 0.1%, suggesting a greater resistance of this species to MPs. The 1% MP concentration caused a decrease in respiration, but the effect was less pronounced than in other species, probably due to the fact that *L. terrestris*, as an anecic species (deep burrowing), may have a better ability to avoid microplastics in the soil, which explains the smaller impact of MPs on its metabolism. For *D. veneta*, a clear decrease in respiration was observed at 1% MP, which indicates a toxic effect, while 0.1% MP caused only minor changes, suggesting moderate resistance in this species. The strongest decrease in respiration among all species was observed for *A. caliginosa*. The 1% MP concentration reduced respiration in this species by more than half, suggesting a very strong toxic effect. A probable explanation is that *A. caliginosa*, as an endogeic species (living in mineral soil), may have greater contact with microplastics, which may increase the negative impact of MPs.

**Figure 4:**
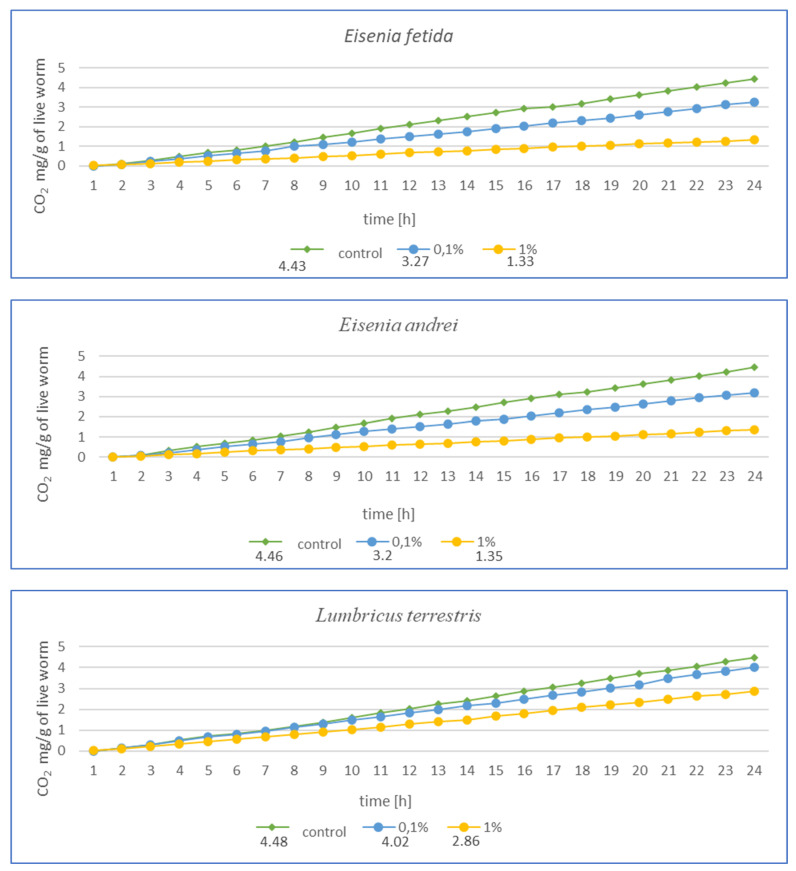

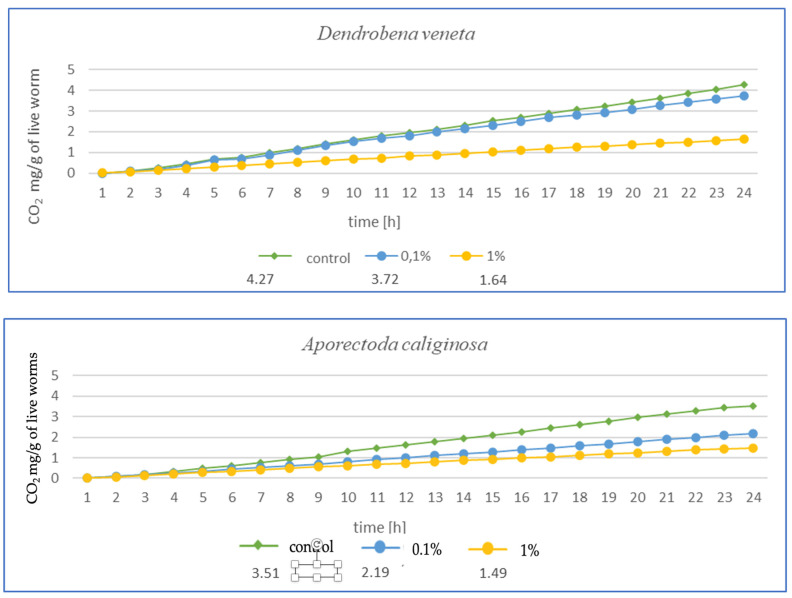
Effects of microplastics on respiration in tested earthworms after 3 months of exposure; mg CO_2_ emitted per g of live worms and per hour.

After 3 months of exposure (Figure 4) for *E. fetida*, a significant decrease in respiration was noted at 1% MPs, suggesting a strong inhibition of metabolism, while at 0.1% MPs, the effect was less pronounced but still present. For *E. andrei*, a similar trend was observed as for *E. fetida*—a strong decrease in respiration at 1% MPs, while 0.1% MPs reduced CO_2_ emission, but not as drastically, which suggests lower toxicity at this concentration. The smallest effect of MPs at 0.1% was noted for *L. terrestris*, which suggests greater resistance in this species. On the other hand, 1% MPs caused a decrease in respiration, but less drastically than in *E. fetida* or *E. andrei.* For the species *D. veneta*, a high decrease in respiration was noted at 1% MPs, which indicated a toxic effect. Additionally, a dose of 0.1% MPs caused only minor changes, which may suggest moderate resistance to low concentrations of MPs. In this study, *A. caliginosa* showed the strongest decrease in respiration among all species. Even at 0.1% MPs, there was a significant reduction in respiration, which indicates high susceptibility to microplastics.

The results after 1 (Figure 3) and 3 months (Figure 4) show a similar trend. As the authors suggest, the MPs used in this study during tissue analysis were found in the septa and respiratory canals of the exoderm, indicating the likelihood of gas exchange disruption at early stages. The most vulnerable are smaller individuals and smaller species, in which a fraction of the size 36–52 µm may have completely blocked gas flow. In the case of *L. terrestris*, no significant changes were observed, and measurements were similar to those obtained from control samples. As for the other studies, the strongest effect was observed in *A. caliginosa*, and this probably correlates with the other negative effects of the presence of plastic in the soil such as oxidative stress and the disruption of the bacterial flora. On a percentage scale, the effects of respiration are shown in the table (Table 1), where the percentage difference in respiration was determined for individuals exposed to particular concentrations of MPs.

The results presented in Table 1 in a percentage manner illustrate the scale of the influence of MPs on gas exchange. Large changes are visible in the case of *A. caliginosa*, which is characterized by high sensitivity to oxidative stresses, as described by biomarkers and gas exchange studies.

### 3.3. Effects on Individual Survival

**Figure 5:**
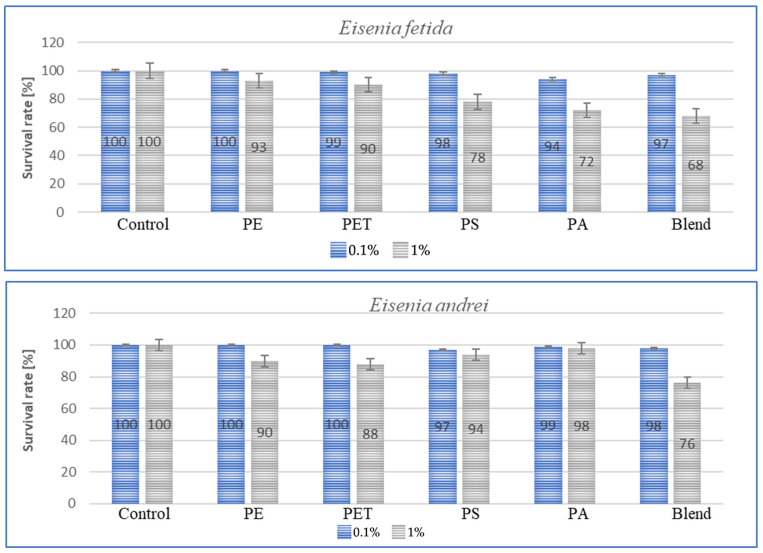

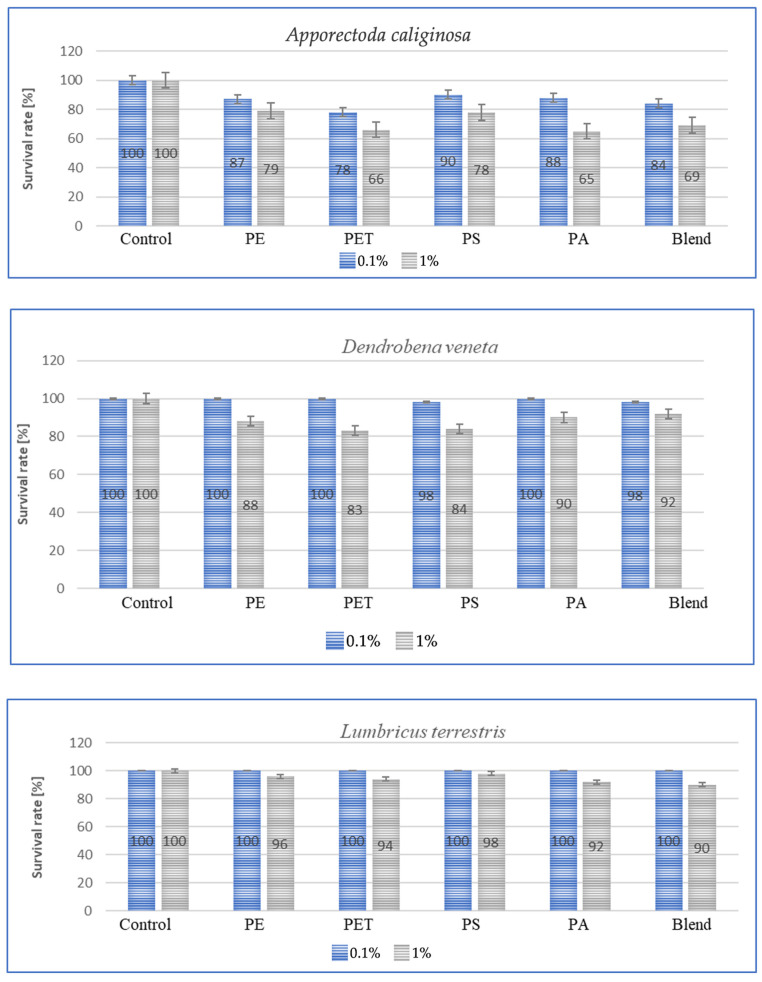
Effect of microplastics on individual survival in tested earthworms.

Species sensitivity is usually defined as the ability to adapt to changing environmental or living conditions. This sensitivity can be expressed in the amount of individual weight lost, concentration, amount of immune proteins and species survival rate. Survival, in turn, along with other parameters, can be used for species bioindication and suggest a foundation for further work aimed at comparing the results obtained with stress responses such as those associated with the presence of heavy metals or varying soil temperatures. The results of the tests conducted to determine survival rates are shown in the graphs below for each species after a period of 3 m. The graphs (Figure 5) show species survival rates after a period of 3 m in earthworms exposed to microplastics contained in the soil.

For all individuals, a small negative effect of high MP concentrations on species survival was observed, and for dose of 1%, the effect was significant, with individual losses being highest in *A. caliginosa* for all tested microplastics materials. The exposure of *E. fetida* to PS, PA and Blend caused a significant decrease in survival rate (Figure 5). Also, for Blend, the microplastic mixture, a negative effect on survival was also noted for *E. andrei* (Figure 5). The species in which survival was the highest were *D. veneta* and *L. terrestris*, where over 95% individual survival was recorded at a concentration of 0.1%. The general trend among publications is similar, where MPs at low concentrations have little effect on individual survival and only high concentrations contribute to an increase in the mortality rate.

### 3.4. Effects of Microplastics on Bacterial Flora

The bacterial flora of the earthworm gut is diverse with respect to its functions and actively supports both digestive processes and the transformation of mineral particles in the soil. This study compared the rate of bacterial flora growth on selected media for each type of plastic and selected earthworm species (Table 2 and Table 3).

Our study did not examine the mechanical damage caused by plastic particles or the detailed impact on the abundance of specific bacterial groups and gene sequences. Here, the goal was to illustrate the overall effect of MPs on the growth of the collected bacterial flora in optical and quantitative terms. This study showed that individuals of the species *A. caliginosa* were most affected by the death of bacterial flora (Table 2, MacConkey agar and Table 3 LB agar), as the number of bacteria on both media decreased significantly. For *E. fetida* and *E. andrei*, the number of bacteria grown on MacConkey medium in microplastic treatments was also lower compared to that in control treatments. Species in which changes were not observed were *D. veneta* and *L. terrestris* on MacConkey medium, indicating the lack of correlation of microplastics with these species’ bacterial flora. When using LB medium, a decrease in total bacteria was observed for all studied earthworm species (Table 3). This decrease was especially found for the 1% microplastic dosage.

## 4. Discussion

Earthworms are a widespread group of organisms used in many tests and studies, and research on biomass and tissue chemical constituents [22], the impact of heavy metals [23] and their importance in vermicomposting [24], among other topics, has been described. They are an extremely important part of the soil ecosystem and are bioindicators for many contaminants [25]. Increasingly, earthworms are also becoming the subject of studies on microplastics and total plastic contaminants in soil [26]. A number of negative effects have been described, manifested by, among other things, a decrease in earthworm weight [27], oxidative stress and increased individual or species mortality [28]. In these studies, *E. fetida* and *E. andrei* are the most commonly described species, while species found in forested areas are studied less frequently.

### 4.1. Biotic Response of Earthworms to MPs 

In our study, we used five different species (*L. terrestris*, *D. veneta*, *A. caliginosa*, *E. fetida*, *E. andrei*), which were subjected to experiments on the effects of microplastics on selected biochemical parameters under laboratory conditions. Oxidative stress, the effects of microplastics on gas exchange, species survival and the effects of microplastics on bacterial flora were examined, and all experiments were carried out at two time intervals (one and three months) and at two concentrations of microplastics. In addition, this study used a mixture of microplastic fractions in addition to individual fractions for a better representation of natural contamination, representing a novel approach.

In the case of stress and toxicity level, all specimens and all species tested showed symptoms of stress at varying levels, which is consistent with many publications [29,30], with *A. caliginosa* and *E. fetida* being most affected by oxidative stress after one month of exposure. The effects of MPs were species, polymer and concentration dependent, suggesting that different MPs may have different mechanisms of action in soil. The high GST values for 1% MPs show that the effects of microplastics may be dose dependent, highlighting the need for further research into toxicity thresholds for soil organisms. Polystyrene (PS) and polyamide (PA) showed the highest GST activity in the pre-test experiments after 3 m in most species, which indicates their stronger effect on oxidative stress in the long term. The microplastic blend (Blend) was very toxic to *A. caliginosa* and *D. veneta*, but its effect was reduced for *E. fetida.*

This study showed that the mixed microplastic fraction had the greatest effect on adaptative biomarkers, levels of which were highest in *A. caliginosa*. The MP concentration was of key importance, and in each case, it altered the level of stress markers. Similar results were obtained by Rodrogez et al. [18], who described the effects of microplastics on the species *E. fetida*, confirming that only high concentrations of microplastics in the soil led to significant changes, as well as Baloš [31]; references for the effects of microplastics on earthworms can also be found in articles by Lackman [32], Baeza [33], Huerta [34] and Dąbrowska [35].

These results were similar to those described after three months of exposure, and in both cases, both *L. terrestris* and *D. veneta* exhibited higher stress resistance. This is likely related to the size of the individuals themselves; the *L. terrestris* individuals used in this study were several times larger than the *Eisenia* individuals. When it comes to determining the effects of microplastics on respiration, this is one of the first studies documenting the effect of microplastics on gas exchange. Our research has shown that microplastics present in soil significantly reduce gas exchange, despite the fact that the exact mechanisms of this effect are not known. This may be related to plastic particles that adhered to the body or were taken up by earthworms, which, by piercing tissue barriers, blocked gas exchange in body septa. In all species and combinations studied, the effect of 1% microplastics drastically affected gas exchange, but in the case of 0.1%, the differences were not as significant, especially in the case of *L. terrestris* and *D. veneta*. There is ample evidence of close links between gas exchange and other metabolic systems in earthworms, such as decreased gas exchange during estivation, the effect of temperature on metabolism and gas exchange and interspecies differences in metabolism and gas exchange [3,24]. However, these factors were not obvious in the present study. A significant negative effect on respiration was observed in earthworms exposed to microplastics. There is currently a lack of direct scientific studies examining the effects of microplastics on earthworm respiratory processes. However, there are reports indicating a general negative impact of microplastics on the health of these organisms, which may affect their general condition and metabolic functions. Although another study [9] does not directly address respiratory processes, it suggests that the presence of microplastics may affect the general health and metabolism of earthworms, which in turn may affect their respiratory functions. However, further targeted studies are needed to precisely determine the effects of microplastics on the respiratory processes of these organisms.

The authors of this study suggest that more detailed studies describing tissue histopathology and the exact sites of microplastic content should be conducted, as demonstrated by the authors of another publication [36]. Additionally, in the case of the mixed fraction, the exact type of plastic that may have caused the observed effects should be determined. One study [17] indicated the impact of microplastics on the skin of earthworms. This study revealed that exposure to microplastics leads to damage on the skin surface of earthworms, which may affect their health and physiological functions. Although this study did not directly examine anatomical differences by habitat, the results suggest the need for further research on the impact of microplastics on different species of earthworms, which may have different skin adaptations depending on their habitat. The study conducted by [10] does not directly focus on skin changes or anatomical differences, but it does provide context for potential research on the interactions between microplastics and earthworm anatomy in different habitats. The impact of microplastics on earthworm tissue surfaces is an under-researched area.

Another study [28] was also conducted on the effect of polystyrene microplastics (PS-MPs) on earthworms of the species *E. fetida*. Histopathological analysis showed damage to intestinal cells in earthworms exposed to PS-MPs, indicating a toxic effect of these particles on gastrointestinal tissues. Exposure to PS-MPs caused significant changes in the levels of glutathione (GSH) and superoxide dismutase (SOD) activity in earthworm tissues. These changes suggest that PS-MPs induce oxidative stress, which may lead to further cellular damage. Our research also confirmed the presence of oxidative stress in earthworm tissues exposed to microplastic, especially at a dose of 1%. Some toxic effects can be also the result of additives used in microplastic production, like PCB, phthalates and bisphenol A.

The gut bacterial flora of earthworms is diverse, and studies to determine bacterial clusters or their growth have included acid bacteria (*Acidobacteria*, *Coribacteraceae*), archaea (*Crenarchaeota)*, Gram-negative bacteria (*Errucomicrobia*) and aerobic bacteria (*Chitinophagaceae*), as well as *Hyphomicrobiaceae* and environmental bacteria (ubiquitous) such as *Flavobacteriaceae* [37].

The present study on the bacterial flora of earthworms was mainly aimed at visually illustrating the changes occurring in the gut of earthworms from the perspective of basic microbiological media and comparing the results for all species. In this way, it was possible to make a preliminary determination of the differences in the bacterial flora without describing the colonies in detail or characterizing them in relation to their generic affiliations. This provided results that were clear and readable from the perspective of researchers not involved in microbiology but in the area of interest of earthworm physiology and changes in intestinal flora. In the case of Gram-negative lac+ and lac− bacteria for *E. fetida* and *E. andrei*, a documented increase in intestinal sterility was evident, but it was not as critical as in the case of *A. caliginosa;* in the case of this species, a strong bacterial reduction was observed, which certainly translated to changes in individual weight, oxidative stress markers and other biochemical parameters. In the case of *L. terrestris* and *D. veneta*, no changes were observed, which may suggest no effect of the microplastics on bacterial flora. For the total number of bacteria (LB medium), the changes were much more intensive, indicating the negative effect of applied microplastics, especially at a dose of 1%, on the bacterial flora of tested earthworms. Lear et al. indicated that microplastic ingestion may lead to dysbiosis of the gut microbiota, which may differ between worm species. Differences in microbiome composition may affect the mechanisms of microplastic uptake and metabolism, suggesting the need for further research into species-specific responses, which was partly confirmed in the present study [11]. A snapshot of available data shows that microplastics can affect heavy metal accumulation by reducing carbohydrate and amino acid metabolism needed for bacterial growth [38].

### 4.2. Ecological Effects

The effects of microplastics on survival described in this study provide a basis for analysis relative to adaptation, but some limitation here may be the small size of the containers and the lack of mobility of the earthworms. Considering their ability to actively move and tunnel, the 3 L containers seem small here, as the study only aimed to show interspecies differences and confirm them based on the available literature. Many publications describe a decrease in earthworm weight, a decrease in cocoons and increased mortality [13,39], but there is not much information on the effect of the mixed microplastic fraction or the comparison of data for several different species with variable environmental adaptations. Our study showed very high survival rates for species exposed to 0.1% microplastics in soil, but at 1%, the changes observed were significant. The species most susceptible to microplastics were *E. andrei* and *E. fetida*, but notably, the highest impact on decreasing survival rate was noticed for *A. caliginosa*, where the most negative changes occurred and the mortality rate was significant. Similar results and descriptions were shown in studies by Cao [40], Zhang [41] and Wang [42], among others.

A study by Boots et al. [9] showed that the presence of microplastics in the soil negatively impacts earthworm health, leading to weight loss and reduced growth. These observations suggest that microplastics may cause irritation and blockages in the earthworms’ digestive tracts, which limits nutrient absorption and affects their fitness. In addition, changes in soil pH caused by microplastics may affect different species of earthworms differently, affecting their ability to assimilate these particles. The mechanisms of response to microplastics may be similar to those observed in aquatic worms studied previously. The effects of plastics include irritation and obstruction of the gastrointestinal tract, reduced nutrient absorption and reduced growth [9]. Judy et al. [43] found no evidence of any effect of microplastics on wheat seedling emergence and production or on mortality or population performance in earthworms and nematodes, which is inconsistent with the results obtained in this study. In the present study, all studied species showed a decrease in CO_2_ emissions after exposure to MPs, which indicates reduced metabolic activity and the potential weakening of vital functions. The species most susceptible to microplastics, such as *A. caliginosa* and *E. fetida*, showed the strongest decrease in respiration, which suggests their high susceptibility to the toxic effects of MPs. On the other hand, *L. terrestris* showed the weakest effect of MPs on metabolism, which suggests a greater resistance of this species to this contamination. The decrease in CO_2_ emissions was strongly dependent on the concentration of microplastics—the effect was more pronounced at 1% MP than at 0.1% MPs. Epigeic species (*E. fetida*, *E. andrei*) and endogeic species (*A. caliginosa*) are most susceptible to microplastics, while anecic species (*L. terrestris*) are more resistant. Reduced earthworm activity may have a negative impact on soil ecosystems, as these organisms play a key role in matter cycling and soil aeration.

### 4.3. Microplastic Parameters and Pollution at Terrestrial Sites

The present study does not provide direct information on how the size of microplastics affects earthworms, since the microplastics exhibited quite similar particle sizes: PE 1–63 µm, PET 10–100 µm, PA 10–200 µm, PS 20–100 µm. Researchers [18] examined the effects of the exposure of earthworms (*E. fetida*) to low-density polyethylene (LDPE) microplastics with sizes ranging between 250 and 1000 μm, but at different doses. There are few studies that report effects in relation to different sizes of a specific single microplastic. In one study [44], the diameters of the PE microplastics were 180–212 μm and 250–300 μm. It was found that both negligibly affected female earthworm reproductive organs but damaged male reproductive organs. The size-dependent toxicity of microplastics was not observed due to similar size of the MPs used, as well as limitations in applying MPs not in a range of sizes but of a specific size. This effect could be caused by small differences in size, but researchers have proven the formation of plastic nanoparticles and their presence in tissues. The polymers selected for this study—polyethylene (PE), polyethylene terephthalate (PET), polystyrene (PS) and polyamide (PA)—are commonly found in terrestrial ecosystems. Their presence results from the widespread use of these materials in everyday products and from improper disposal of plastic waste [45].

Quality assurance and sample quality control (QA/QC) in microplastic testing are key to minimizing sample contamination and obtaining reliable and repeatable results. The use of rigorous QA/QC procedures, such as the use of clean reagents, blank controls and the avoidance of cross-contamination, allows for the accurate identification and quantification of microplastics in environmental samples [46].

## 5. Conclusions

While there are many specialized reports on the effects of plastic on earthworms, this article is one of the few to compare both different species and a mixed fraction of microplastics, which is new to the literature. The results of this study provide a better understanding of interspecies differences and adaptation to changing habitat conditions.

Full agreement remains to be achieved, however, on the species best adapted to plastic exposure. According to the authors’ assumptions, *D. veneta*, as a species often found in urban areas, urbanized spaces and contaminated areas, tolerated the presence of plastic in the soil very well, showing no morphological–chemical changes at a concentration of 0.1%. *L. terrestris*, as a species that reacts similarly to microplastics, probably owes its resistance to individual size, so that plastic fragments that would cause damage in other species remained harmless for this species. For all species, the 1% concentration proved toxic and definitely affected individuals, which is an important clue, as is information that in heavily polluted industrial areas and near illegal landfills, the soil may be devoid of earthworms. This study on the impact of microplastics (MPs) on earthworms showed significant differences in responses among species, which is likely due to different ecological niches, foraging patterns and tunneling strategies. Epigeic species, such as *E. andrei* and *E. fetida*, are more exposed to direct MP ingestion, leading to increased oxidative stress and changes in the gut microbiome. Endogeic and anecic species, such as *A. caliginosa* and *L. terrestris*, face longer-term exposure to MPs, which may result in chronic effects on their metabolism and survival. Variability in the response to MPs may be due to differences in the metabolism and detoxification capacity of individual species. Identifying species most susceptible to MP contamination can help to better monitor the environmental status and assess the effects of plastic pollution. The conducted study confirms that earthworms are exposed to oxidative stress caused by microplastics, which may affect their health and ecosystem functions. This study examined the impact of MPs on different species, and the results suggest that soil ecosystems may be changed by the long-term accumulation of microplastics. Therefore, identifying species more susceptible to microplastics may help to define bioindicator organisms for assessing environmental contamination. Microplastics can disrupt earthworm metabolism through oxidative stress, changes in the microbiome and reduced nutrient absorption. In the present study, it was found that earthworms exposed to microplastics show reduced respiration, which indicates reduced metabolic activity.

## Figures and Tables

**Table 1:** Decrease in gas exchange efficiency after one and three months compared to the control sample.

Species	Concentration (*w*/*v*%)	1 Month	3 Months
*E. andrei*	0.1% 1%	−19.56% −38.69%	−28.25% −69.73%
*E. fetida*	0.1% 1%	−22.58% −40.13%	−26.18% −69.97%
*A. caliginosa*	0.1% 1%	−37.60% −57.54%	−46.00% −60.32%
*L. terrestris*	0.1% 1%	no changes −28.57%	−10.26% −36.16%
*D. veneta*	0.1% 1%	−13.47% −37.82%	−12.88% −61.59%

**Table 2:** Differences in bacterial flora cultures on MacConkey medium after one and three months of exposure to MPs.

	Control	1 Month 0.1%	3 Months 1%
*E. fetida*	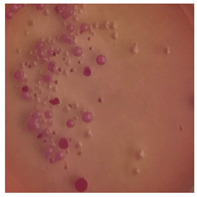	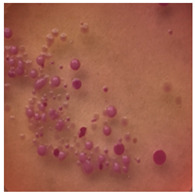	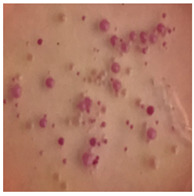
*E. andrei*	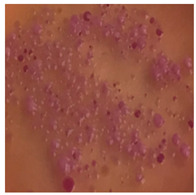	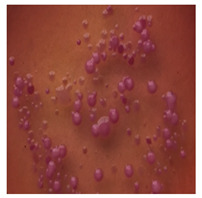	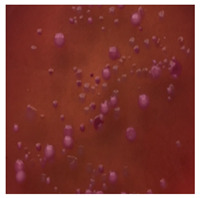
*A. caliginosa*	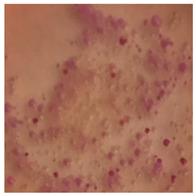	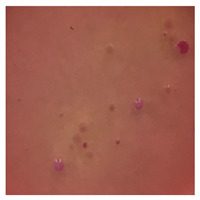	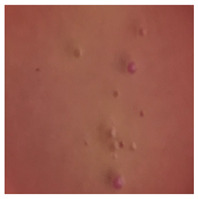
*D. veneta*	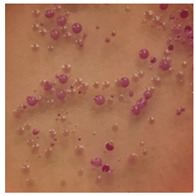	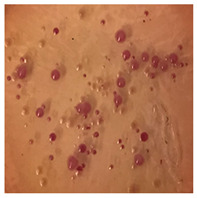	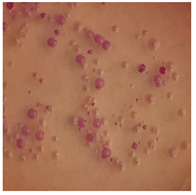
*L. terrestris*	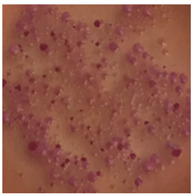	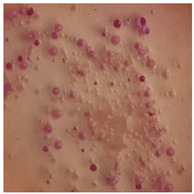	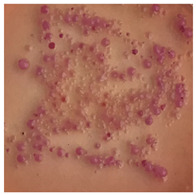

**Table 3:** Differences in bacterial flora cultures on LB agar medium after one and three months of exposure to MPs.

	Control	1 Month 0.1%	3 Months 1%
*E. fetida*	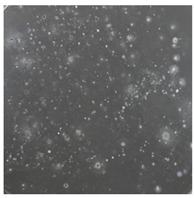	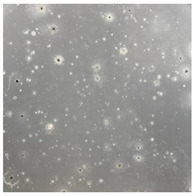	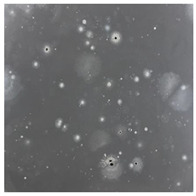
*E. andrei*	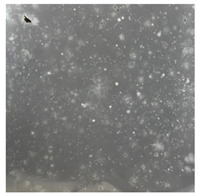	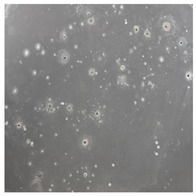	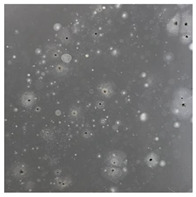
*A. caliginosa*	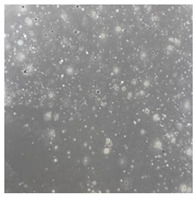	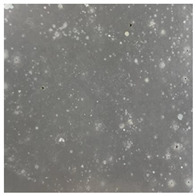	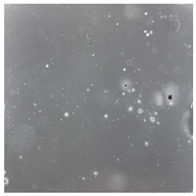
*D. veneta*	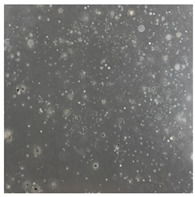	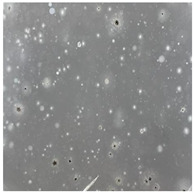	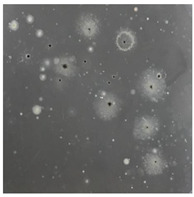
*L. terrestris*	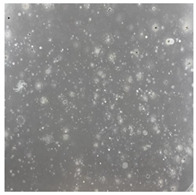	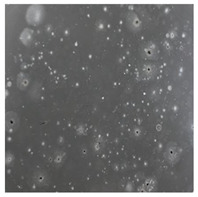	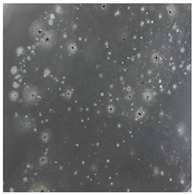

## Data Availability

The data that support the findings of this study are available from the corresponding author upon reasonable request.

## References

[1] Briones M.J.I., Moran P., Posada D. (2009). Are the sexual, somatic and genetic characters enough to solve nomenclatural problems in lumbricid taxonomy?. Soil Biol. Biochem..

[2] Csuzdi C., Zicsi A. (2003). Earthworms of Hungary (Annelida: Oligochaeta, Lumbricidae).

[3] Adhikari K., Astner A.F., DeBruyn J.M., Yu Y., Hayes D.G., O’Callahan B.T., Flury M. (2023). Earthworms Exposed to Polyethylene and Biodegradable Microplastics in Soil: Microplastic Characterization and Microbial Community Analysis. ACS Agric. Sci. Technol..

[4] Song J., Chen X., Li S., Tang H., Dong S., Wang M., Xu H. (2024). The environmental impact of mask-derived microplastics on soil ecosystems. Sci. Total Environ..

[5] Suleiman H., Rorat A., Grobelak A., Grosser A., Milczarek M., Płytycz B., Kacprzak M., Vandenbulcke F. (2017). Determination of the performance of vermicomposting process applied to sewage sludge by monitoring of the compost quality and immune responses in three earthworm species: *Eisenia fetida*, Eisenia andrei and Dendrobaena veneta. Bioresour. Technol..

[6] Cole M., Lindeque P., Halsband C., Galloway T.S. (2011). Microplastics as contaminants in the marine environment: A review. Mar. Pollut. Bull..

[7] Ivar do Sul J.A., Costa M.F. (2014). The present and future of microplastic pollution in the marine environment. Environ. Pollut..

[8] Zubris K.A.V., Richards B.K. (2005). Synthetic fibers as an indicator of land application of sludge. Environ. Pollut..

[9] Barceló D. (2024). Microplastics in the environment: Analytical chemistry methods, sorption materials, risks and sustainable solutions. Anal. Bioanal. Chem..

[10] de Souza Machado A.A., Lau C.W., Till J., Kloas W., Lehmann A., Becker R., Rillig M.C. (2018). Impacts of microplastics on the soil biophysical environment. Environ. Sci. Technol..

[11] Lear G., Kingsbury J.M., Franchini S., Gambarini V., Maday S.D.M., Wallbank J.A., Weaver L., Pantos O. (2021). Plastics and the microbiome: Impacts and solutions. Environ. Microbiome.

[12] Kallenbach E.M., Rødland E.S., Buenaventura N.T., Hurley R. (2022). Microplastics in terrestrial and freshwater environments. Microplastic in the Environment: Pattern and Process.

[13] Liu Y., Xu G., Yu Y. (2022). Effects of polystyrene microplastics on accumulation of pyrene by earthworms. Chemosphere.

[14] Thompson R.C., Moore C.J., vom Saal F.S., Swan S.H. (2009). Plastics, the environment and human health: Current consensus and future trends. Philos. Trans. R. Soc. B.

[15] Wang J., Coffin S., Sun C., Schlenk D., Gan J. (2019). Negligible Effects of Microplastics on Animal Fitness and HOC Bioaccumulation in Earthworm Eisenia Fetida in Soil. Environ. Pollut..

[16] Lwanga E.H., Gertsen H., Gooren H., Peters P., Salánki T., van der Ploeg M., Besseling E., Koelmans A.A., Geissen V. (2017). Incorporation of microplastics from litter into burrows of Lumbricus terrestris. Environ. Pollut..

[17] Trakić T., Popović F., Sekulić J., Hackenberger D.K. (2024). Ecotoxicological Effects of Commercial Microplastics on Earthworm *Eisenia fetida* (Savigny, 1826) (Clitellata; Lumbricidae). Agriculture.

[18] Rodríguez-Seijo A., da Costa J.P., Rocha-Santos T., Duarte A.C., Pereira R. (2018). Oxidative stress, energy metabolism and molecular responses of earthworms (*Eisenia fetida*) exposed to low-density polyethylene microplastics. Environ. Sci. Pollut. Res..

[19] Habig W.H., Pabst M.J., Fleischner G., Gatmaitan Z., Arias I.M., Jakoby W.B. (1974). The Identity of Glutathione *S*-Transferase B with Ligandin, a Major Binding Protein of Liver. Proc. Natl. Acad. Sci. USA.

[20] Hugo A. (1984). Catalase in vitro. Methods in Enzymology.

[21] Marion B. (1976). A rapid and sensitive method for the quantitation of microgram quantities of protein utilizing the principle of protein-dye binding. Anal. Biochem..

[22] Kostecka J., Garczyńska M., Pączka G., Mazur-Pączka A. (2022). Chemical composition of earthworm (*Eisenia fetida* Sav.) biomass and selected determinants for its production. J. Ecol. Eng..

[23] Kowald G.R., Stürzenbaum S.R., Blindauer C.A. (2016). Earthworm *Lumbricus rubellus* MT-2: Metal Binding and Protein Folding of a True Cadmium-MT. Int. J. Mol. Sci..

[24] Dominguez J., Gomez-Brandon M. (2013). The influence of earthworms on nutrient dynamics during the process of vermicomposting. Waste Manag. Res..

[25] Stürzenbaum S.R., Kille P., Morgan A.J. (1998). The identification, cloning and characterization of earthworm metallothionein. FEBS Lett..

[26] Khalid N., Aqeel M., Noman A. (2020). Microplastics could be a threat to plants in terrestrial systems directly or indirectly. Environ. Pollut..

[27] Huang Y., Liu Q., Jia W., Yan C., Wang J. (2020). Agricultural plastic mulching as a source of microplastics in the terrestrial environment. Environ. Pollut..

[28] Jiang X., Chang Y., Zhang T., Qiao Y., Klobučar G., Li M. (2020). Toxicological effects of polystyrene microplastics on earthworm (*Eisenia fetida*). Environ. Pollut..

[29] Cheng Y., Zhu L., Song W., Jiang C., Li B., Du Z., Wang J., Wang J., Li D., Zhang K. (2020). Combined effects of mulch film-derived microplastics and atrazine on oxidative stress and gene expression in earthworm (*Eisenia fetida*). Sci. Total Environ..

[30] Kadac-Czapska K., Ośko J., Knez E., Grembecka M. (2024). Microplastics and Oxidative Stress—Current Problems and Prospects. Antioxidants.

[31] Baloš M., Petrović A., Tubić A., Zeremski T., Gvozdenac S., Supić D., Bursić V. (2024). Effects of Polyethylene Microplastics in Agricultural Soil on *Eisenia fetida* (Annelida: Oligochaeta) Behavior, Biomass, and Mortality. Agriculture.

[32] Lackmann C., Velki M., Šimić A., Müller A., Braun U., Ečimović S., Hollert H. (2022). Two types of microplastics (polystyrene-HBCD and car tire abrasion) affect oxidative stress-related biomarkers in earthworm *Eisenia andrei* in a time-dependent manner. Environ. Int..

[33] Baeza C., Cifuentes C., Gonzalez P., Araneda A., Barra R. (2020). Experimental exposure of *Lumbricus terrestristo* microplastics. Water Air Soil Poll..

[34] Huerta Lwanga E., Gertsen H., Gooren H., Peters P., Salánki T., Van Der Ploeg M., Besseling E., Koelmans A.A., Geissen V. (2016). Microplastics in the Terrestrial Ecosystem: Implications for Lumbricus terrestris (Oligochaeta, Lumbricidae). Environ. Sci. Technol..

[35] Dabrowska A. (2022). Soil microplastics—Current research trends and challenges: Preliminary results of the earthworm *Eisenia fetida* impact on glitters. Acta Hort. Regiotec..

[36] Klimasz M., Grobelak A. (2024). Accumulation of Spherical Microplastics in Earthworms Tissues-Mapping Using Raman Microscopy. Appl. Sci..

[37] Luyts K., Napierska D., Nemery B., Hoet P.H.M. (2012). How physico-chemical characteristics of nanoparticles cause their toxicity: Complex and unresolved interrelations. Environ. Sci. Process. Impacts.

[38] Yang Y., Xu G., Yu Y. (2022). Microplastics impact the accumulation of metals in earthworms by changing the gut bacterial communities. Sci. Total Environ..

[39] Zaller J.G., Saxler N. (2007). Selective vertical seed transport by earthworms: Implications for the diversity of grassland ecosystems. Eur. J. Soil Biol..

[40] Cao D., Wang X., Luo X., Liu G., Zheng H. (2017). Effects of Polystyrene Microplastics on the Fitness of Earthworms in an Agricultural Soil. IOP Conf. Ser. Earth Environ. Sci..

[41] Zhang L., Sintim H.Y., Bary A.I., Hayes D.G., Wadsworth L.C., Anunciado M.B., Flury M. (2018). Interaction of Lumbricus Terrestris with Macroscopic Polyethylene and Biodegradable Plastic Mulch. Sci. Total Environ..

[42] Li W., Zu B., Li L., Li J., Li J., Mei X. (2023). Desorption of bisphenol A from microplastics under simulated gastrointestinal conditions. Front. Mar. Sci..

[43] Judy J.D., Williams M., Gregg A., Oliver D., Kumar A., Kookana R., Kirby J.K. (2019). Microplastics in municipal mixed-waste organic outputs induce minimal short to long-term toxicity in key terrestrial biota. Env. Pollut..

[44] Kwak J.I., An Y.J. (2021). Microplastic digestion generates fragmented nanoplastics in soils and damages earthworm spermatogenesis and coelomocyte viability. J. Hazard. Mater..

[45] Schell T., Rico A., Vighi M. (2020). Occurrence, fate and fluxes of plastics and microplastics in terrestrial and freshwater ecosystems. Rev. Environ. Contam. Toxicol..

[46] Taurozzi D., Gallitelli L., Cesarini G., Romano S., Orsini M., Scalici M. (2024). Passive biomonitoring of airborne microplastics using lichens: A comparison between urban, natural and protected environments. Environ. Int..

